# Surgeon Perspectives on Descemetorhexis Without Endothelial Keratoplasty for Fuchs Endothelial Corneal Dystrophy: A UK National Survey

**DOI:** 10.7759/cureus.72899

**Published:** 2024-11-02

**Authors:** Gagandeep S Sachdeva, Riddhi Thaker, Stella Hristova, Ahmed Bardan

**Affiliations:** 1 Postgraduate Medical Education, Royal Wolverhampton NHS Foundation Trust, Wolverhampton, GBR; 2 Ophthalmology, East Suffolk and North Essex NHS Foundation Trust, Colchester, GBR; 3 Ophthalmology, Leeds Teaching Hospitals NHS Trust, Leeds, GBR; 4 Ophthalmology, Leeds University Hospitals NHS Foundation Trust, Leeds, GBR

**Keywords:** corneal transplantation, descemet membrane endothelial keratoplasty, descemetorhexis without endothelial keratoplasty, fuchs endothelial dystrophy, keratoplasty

## Abstract

Introduction: To provide an insight into the current perspective of UK ophthalmic surgeons on the role of Descemetorhexis without endothelial keratoplasty (DWEK) for the management of Fuchs endothelial corneal dystrophy (FECD).

Materials and methods: A Google Form (Google, UK) was electronically distributed to UK ophthalmologists with a special interest in cornea from November 2023 to June 2024. The survey consisted of 13 mandatory questions.

Results: Responses were received from 36 ophthalmic surgeons from across the UK, with clinical experience ranging from 0.5 to 30 years. Descemet membrane endothelial keratoplasty (DMEK) and Descemet stripping (automated) endothelial keratoplasty (DSEK/DSAEK) were the most common approaches for managing FECD. Only four respondents had practiced DWEK, but 97.2% expressed interest in learning this technique (35/36). Additionally, 69.4% felt that DWEK would be beneficial only with RHO kinase inhibitors (25/36). All respondents agreed that the clinical field would benefit from randomised trials comparing DWEK to DMEK/DSAEK, and 97.2% were open to changing their clinical practice based on the findings of such trials (35/36).

Discussion: This article provides the first snapshot of UK ophthalmic surgeons’ views on DWEK in FECD. DWEK has potential as a surgical technique in selective cases, particularly with RHO kinase inhibitors. Optimal visual outcomes with DWEK depend on careful patient selection, necessitating an evidence-based approach to define patient selection criteria. Future studies should compare visual and complication outcomes between DWEK and DMEK/DSAEK in clinical trials, incorporating RHO kinase inhibitors.

## Introduction

Fuchs endothelial corneal dystrophy (FECD) is the most prevalent cause of corneal dystrophy, characterised by primary corneal endothelial cell dysfunction. Epidemiological studies suggest it is more common in women with increasing age [1-2]. The loss of corneal endothelial cells is a hallmark of the disease, and at critical endothelial cell count loss, corneal oedema and vision loss can occur.

Techniques of keratoplasty have evolved, and for over a century, penetrating full-thickness grafts were the mainstay treatment option for FECD [3]. The current surgical practice involves partial-thickness selective grafts, with FECD representing the most common indication for corneal transplantation worldwide [4]. Descemet membrane endothelial keratoplasty (DMEK) and Descemet stripping (automated) endothelial keratoplasty (DSEK/DSAEK) are currently the most recognised techniques [5], with literature evidence to suggest superior visual acuity outcomes with DMEK [6]. This has also been suggested as a popular opinion amongst ophthalmic surgeons, despite many currently performing approximately equal numbers of DMEK and DSAEK due to surgical difficulty or being on the learning curve for DMEK procedures [7].

However, selective endothelial keratoplasty techniques are subject to limitations in practice, including the shortage of corneal donors, the requirement for graft preparation and post-operative topical steroids, rebubbling of detached grafts, the risk of infection, rejection, failure, and the expense [8]. A novel technique of Descemetorhexis without endothelial keratoplasty (DWEK) is being studied and suggests scope for overcoming the limitations associated with donor corneal tissue. This procedure involves only the peeling of the central Descemet membrane and corneal endothelium, with preservation of the peripheral endothelium. Specular microscopy has identified the centripetal migration of stem-cell-like cells from the peripheral endothelium into areas of bare posterior stroma [9]. The post-operative utility of RHO kinase inhibitors after DWEK has also been suggested to increase the rates of endothelial cell proliferation and migration, reducing the time to corneal clearance [10]. Initial head-to-head comparative studies between DWEK and DMEK suggest non-inferior visual outcomes with DWEK in patients with mild to moderate FECD, but the time to recovery of vision may be longer [11].

Initial outcome data for DWEK appears promising and the present evidence base would benefit from the prospective study into its therapeutic value for FECD. However, there has been no formal study into the current perspective of ophthalmic surgeons on the role of DWEK. In the present study, we assess the current practice patterns of UK ophthalmic surgeons for the management of FECD and gather their opinions on the potential role of DWEK.

## Materials and methods

The overall aim of the present study was to gauge the knowledge and understanding of the use of DWEK and views held by UK ophthalmic surgeons.

A Google Form (Google, UK) was first electronically distributed to corneal specialist ophthalmic surgeons on 4 November 2023 and responses were accepted until 16 June 2024. Participation in the study was voluntary, and respondents were not required to provide any identifiable information but were able to provide voluntary disclosure of their email addresses to express their interest in participating in future randomised controlled trials (RCTs). A statement of consent was provided, and by completing the survey, respondents consented to their responses being used for data analysis. The data collection for the present review constituted a multi-institutional design, both in terms of authors and survey responders. The authors collaborated virtually and were known to each other from previous clinical and academic work and identified a mutual interest in the topic during the COVID-19 pandemic due to the lack of corneal donor tissue and the emerging novel technique of DWEK which proposed to overcome this limitation. MS Excel (Microsoft Corporation, Redmond, Washington, United States) document was created, dividing the UK by regions and allocating specific regions to a particular author (Hristova, Sachdeva, Thaker). Each region was then searched systematically via a Google UK search to identify the name and contact of corneal specialists by region, using the information that was available in the public domain. This information was tabulated into MS Excel, and the date listed on which an email was sent to the corneal specialist detailing the purpose of our review and a confidential link to the Google Survey form. Only one attempt was made to contact the said designated party. In addition to this systematic screening of UK-based corneal specialists, personally known contacts and affiliations via the Royal College of Ophthalmologists were also contacted to disseminate the Google Survey.

The survey was made up of 13 mandatory questions with a separate section to provide additional comments, where indicated (Appendix 1). The survey collected information on physician’s demographics of clinical practice, experience level, and their current practice patterns for managing FECD. Respondents were then asked for their perspective on the role of DWEK, whether they are currently performing DWEK for FECD, and their opinion on the post-operative utility of RHO kinase inhibitors. Finally, respondents were asked whether further RCT evidence comparing DWEK to DMEK/DSAEK would be of benefit, whether the findings from such a study would change their clinical practice, and whether their Trust would be interested in participating in such a study.

Formulated as a qualitative study design, no formal statistical analysis was required. The data outputs from the survey were collated into MS Excel document to allow common themes to be identified. Numerical counts alone were performed and used as the summary metric for the data, both for baseline demographic data and also specific output data on the perspectives of DWEK. Where specific written comments were of value, these were also considered and extrapolated into the results section. Responses from the survey were tabulated (Appendix 2), analysed, and synthesised into a narrative.

## Results

Thirty-eight survey responses were received, from which 36 were represented within the UK, one from Oman, and one from Mexico. Non-UK-based survey data did not meet the criteria for inclusion in this review, and this was not analysed further. These non-UK-based responses are highlighted separately in Appendix 2. The geographical representation of the responses received was found to be Northern Ireland (5/36), North West (1/36), Yorkshire and the Humber (9/36), Wales (1/36), West Midlands (4/36), East of England (6/36), London (8/36), South East (4/36), and South West (2/36). The 36 UK responses included corneal consultants (28/36), fellows (6/36), and ophthalmology trainees (2/36). Clinical experience levels ranged from 0.5 to 30 years. From this data, one corneal consultant had retired from surgery.

When considering the current practice of respondents for the management of FECD, DMEK was the most common approach (30/36), followed by DSEK/DSAEK (25/36), and penetrating keratoplasty (6/36). One respondent was a corneal consultant who had retired from surgery and as such was not included in the above count of current practice. Four respondents had practiced DWEK (4/36), from which three were corneal consultants with 2.5, 12, and over 20 years of corneal experience, respectively; the fourth was a specialty trainee with one year of corneal experience.

**Figure 1:**
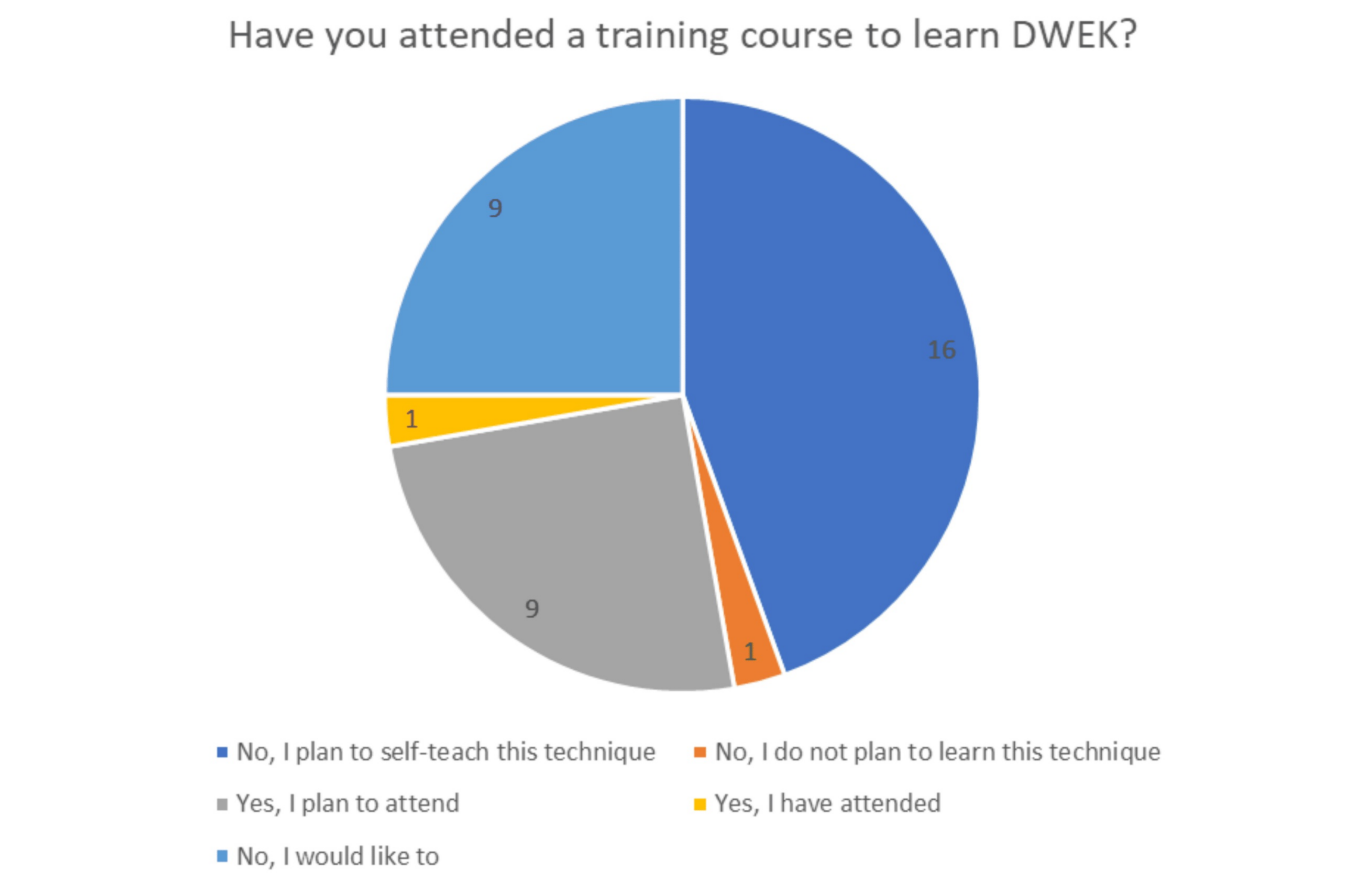
Responses to whether participants have attended a training course for DWEK DWEK: Descemetorhexis without endothelial keratoplasty

Nearly all respondents (35/36) had heard of DWEK as a surgical procedure before the present survey, yet only a single respondent (1/36) had already attended a training course to learn the technique. Half of the respondents either planned to or expressed interest in attending a training course for DWEK (18/36), a significant number planned to self-teach the technique (16/36), and only one respondent did not plan to learn the technique of DWEK (1/36). These findings have been summarised in Figure 1.

**Figure 2:**
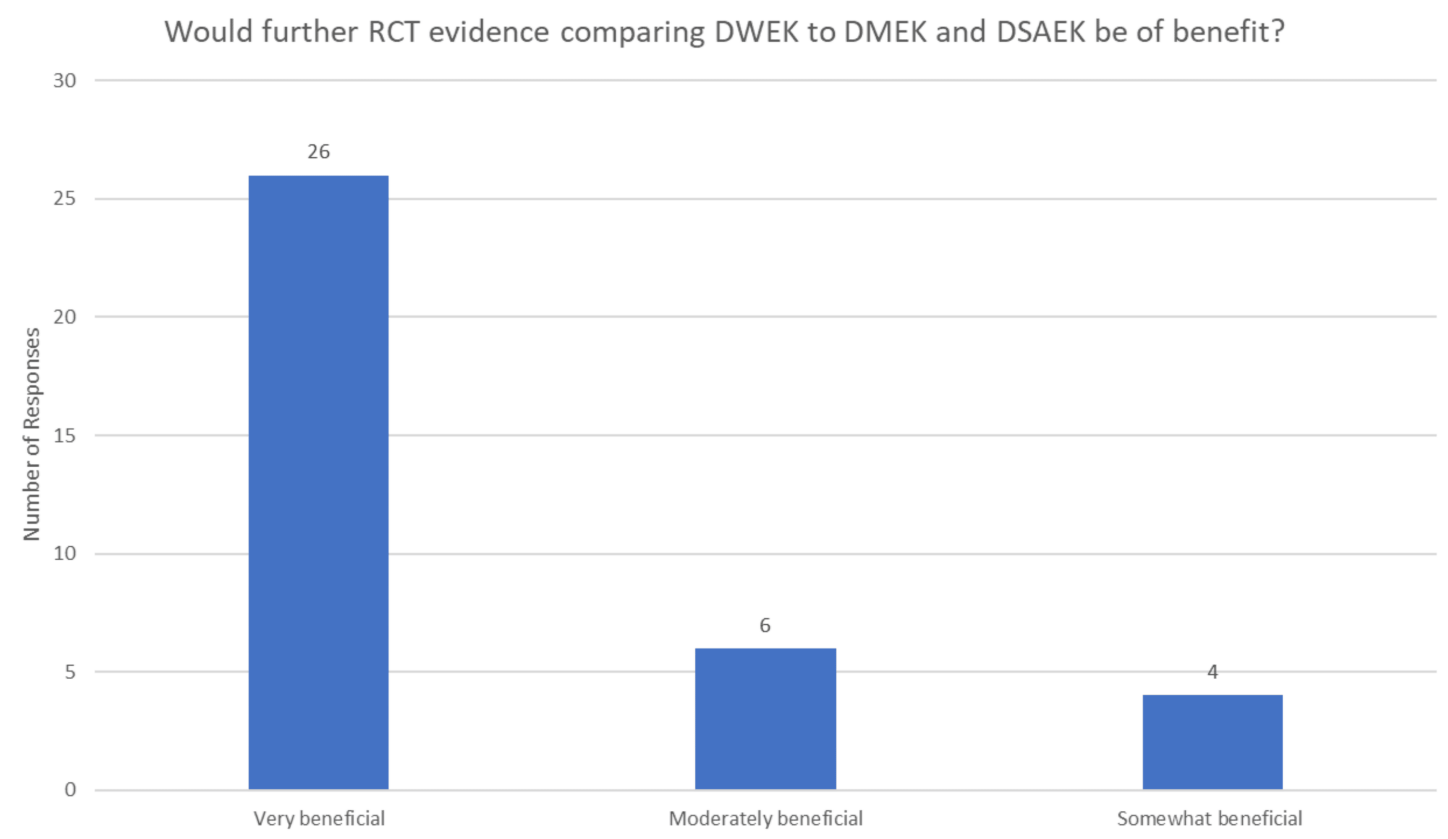
Responses to whether further RCT evidence would be of benefit to compare endothelial keratoplasty techniques RCT: Randomised controlled trial; DWEK: Descemetorhexis without endothelial keratoplasty; DMEK: Descemet membrane endothelial keratoplasty; DSAEK: Descemet stripping (automated) endothelial keratoplasty

All respondents agreed that the clinical study of DWEK comparing this to DMEK/DSAEK in randomised trials would be of benefit in shaping their practice (36/36), with 26/36 defining it as very beneficial, 6/36 defining it as moderately beneficial, and 4/36 defining it as somewhat beneficial. These findings have been summarised in Figure 2. Most respondents also expressed interest in participating in such a randomised study. Most respondents agreed that high-quality evidence from randomised clinical trials would likely change their keratoplasty technique (35/36), with 14/36 defining this as very likely, 16/36 as moderately likely, and 5/36 as somewhat likely. Only one respondent suggested that clinical trial evidence would not be likely to change their practising technique.

Most respondents considered that DWEK could be a suitable technique with RHO kinase inhibitors, with 25/36 respondents identifying that they would not consider DWEK if RHO kinase inhibitors were not accessible at their practicing trust; the remainder describing that they would still consider DWEK if RHO kinase inhibitors were not available (11/36).

## Discussion

This survey reports on the views of UK ophthalmic surgeons on the role of DWEK in FECD in 2024. The data highlights that the respondents were nearly unanimously aware of DWEK, yet only four respondents had practiced this technique. Indeed, the survey data highlights that a paradigm shift in surgical practice would be best driven by evidence-based medicine, with agreement amongst all respondents that the field would benefit from further evidence in randomised trials, comparing DWEK to DMEK/DSAEK.

The study of DWEK at the time of this review largely takes the form of small-scale case reports, case series, and cohort studies [12-16]. There is also limited literature evidence comparing DWEK to DMEK/DSAEK at the time of this review. A popularly cited study at the time of writing included a small-scale retrospective cohort study published in 2018 reporting on 27 eyes, comparing the visual outcomes and associated morbidity of patients with FECD treated with DMEK (15/27) or DWEK (12/27) [9]. Patients with mild to moderate FECD were studied: corneal guttae and oedematous pathology limited to the central cornea, with clear peripheral cornea of limited disease activity. From the studied cohort, equivalent visual outcomes were obtained post-operatively (p = 0.44): Snellen 20/25 -1 (logMAR 0.16 +/- 0.09) for DMEK and Snellen 20/30 +1 (logMAR 0.13 +/- 0.10) for DWEK. Of note, the time to recovery of Snellen 20/40 was significantly shorter in DMEK as compared to DWEK (p < 0.01), with a reported 2.2 +/- 2.8 weeks for DMEK and 7.1 +/- 2.7 weeks for DWEK. The study abstract did not mention the concomitant use of RHO kinase inhibitors.

Interestingly, despite the longer time to recovery with DWEK in this study as reported above, patients receiving DWEK did not experience any adverse events nor the requirement for donor corneal tissue, additional procedures, or long-term immunosuppression. By comparison, eight out of 15 patients in the DMEK group observed adverse events, including increased intraocular pressure, inflammation of the anterior chamber, and non-adherence to the donor graft. This data therefore suggested merit for DWEK as a technique to reduce the overall burden by way of reduced complication rates. This means that patient selection from the severity of FECD to the social impacts of longer recovery is pertinent. The clinical field would benefit from more robust and larger-scale evidence with study into outcome data and complications in clinical trials.

From the FECD cohort, patients should be carefully screened to identify those suitable for DWEK. This is because the procedure involves the central peeling of the Descemet membrane and corneal endothelium, with the subsequent centripetal migration of the preserved peripheral endothelium into areas of bare posterior stroma. It therefore relies on healthy low-disease-activity peripheral endothelium for successful post-operative outcomes. A 2017 paper that studied 11 FECD patients (12 eyes) receiving DWEK defined their exclusion criteria as peripheral endothelial cell counts of <1000 and the presence of central oedema [15]. Two eyes from the studied cohort with stalled endothelial healing at two and three months had the concomitant use of ripasudil Rho kinase inhibitor six times a day for two weeks and observed complete corneal clearance.

These studies highlight the importance of careful patient selection. Patients could be effectively screened with optical slit-lamp microscopy and subsequent imaging including high-definition optical coherence tomography (HD-OCT) for a detailed disease activity assessment. Advances in artificial intelligence, machine learning, and deep learning also suggest scope for automation in staging FECD and quantifying disease activity, helping to streamline the process of patient selection [17-18]. 

To facilitate effective healing and closure of the posterior endothelium with centripetal migration of the peripheral endothelium, RHO kinase inhibitors may be topically instilled after DWEK. RHO kinase inhibitors stimulate endothelial cellular movement, thereby promoting wound closure of the bare endothelium. Animal models have already presented encouraging data on the utility of RHO kinase inhibitors in corneal endothelial wound healing [19-20]. Data from animal models has been supplemented in human studies. One prospective cohort study from 2019 presented data from 18 patients undergoing DWEK and assigned to post-operative ripasudil RHO kinase inhibitor treatment [20]. This study demonstrated quicker vision recovery in patients receiving ripasudil (4.6 weeks vs. 6.5 weeks, p < 0.01) and higher endothelial cell densities at three, six, and 12 months.

However, RHO kinase inhibitors are not currently licensed in the UK for this context. The current scope for RHO kinase inhibitors as defined by the National Institute of Health and Care Excellence (NICE) is in the NG81 Health Technology Appraisal for managing glaucoma [21]. It has been licensed as topical monotherapy or combination therapy in adults with primary open-angle glaucoma or ocular hypertension and elevated intraocular pressures. As licensing agreements are redefined and the availability of RHO kinase inhibitors improves, there could be a good scope to optimise the time to recovery of visual outcomes after DWEK.

Our study benefits from having respondent data from ophthalmic trainees, fellows, and consultants across the UK from numerous practicing hospital trusts. We note that 75.7% of responses received were from corneal consultants (28/36), and the remainder were from trainees and corneal fellows. The present study recognises that specialist trainees are often deeply involved in both patient management and surgery. This is especially true for trainee-selected component (TSC) level trainees in their final or fellowship years, who are often engaged in several cases at a senior level, with some also transitioning into consultant roles within the same discipline. Their invaluable contributions are greatly appreciated in this study, helping to achieve the study aim of gaining a holistic insight into UK surgeons' perspectives of DWEK in FECD.

The main limitation of this study is its sample size of 36 UK-based respondents. In addition to benefiting from more responses overall, the present study would benefit from capturing respondent data from Scotland, North East, and East Midlands.

## Conclusions

Overall, DWEK presents a novel approach to managing FECD, with early evidence suggesting comparable outcomes to the mainstay lamellar technique of DMEK in selective cases. This review represents the first of its kind to provide a snapshot of the views of UK surgeons on the role of DWEK in FECD patients. Our survey data highlights that respondents were keen to develop new proficiency and learn this technique. However, it was unanimously agreed that the field would benefit from further evidence in clinical trials to compare outcome data from DWEK with DMEK and DSAEK. Patients should be carefully assessed for their suitability for DWEK, and the field would benefit from clearly defined patient selection criteria, developed through an evidence-based medicine approach. DWEK should be considered with the concomitant use of RHO kinase inhibitors to promote wound healing and its potential role for deep learning to automate and facilitate the process of patient case selection.

## Appendices

Appendix 1: Google survey template

Surgeon perspectives on Descemetorhexis without endothelial keratoplasty for Fuchs endothelial corneal dystrophy: a UK national survey

About This Survey

The present survey aims to better understand the perspective of UK ophthalmic surgeons on the role of Descemetorhexis without endothelial keratoplasty (DWEK) for the management of Fuchs endothelial corneal dystrophy (FECD). 

Privacy and Confidentiality Statement

The data collected will be anonymised. Respondents are not required to provide any identifiable information but are able to provide voluntary disclosure of their email address to express their interest to participate in future randomised controlled trials (RCTs). 

Consent 

By completing this survey, respondents consent to their responses being used for the purpose of data analysis. The subsequent findings may be shared in reports, presentations, or academic publications. 

Further Queries

Please direct any additional queries relating to this survey to one of the below emails:

Mr. Ahmed Bardan: Ahmed.Bardan@nhs.net

1. Please select the region of your most recent practice (please select all that apply).

Options: Northern Ireland, Scotland, North East, North West, Yorkshire and the Humber, Wales, East Midlands, West Midlands, East of England, London, South East, South West, Other.

2. Which trust do you practice in NHS?

Short answer free text response. 

3. Please indicate your specialty job title. 

Options: Cornea consultant, Trainee, Trust grade, Fellow. 

4. Please indicate how many years you have been practicing in cornea.

Short answer free text response. 

5. What is your current practice for the management of Fuchs endothelial corneal dystrophy? (please select all that apply)

Options: Descemet membrane endothelial keratoplasty (DMEK), Descemet stripping (automated) endothelial keratoplasty (DSEK/DSAEK), Penetrating keratoplasty, DALK, CAIRS, RHO-associated kinase inhibitor eye drops, Other.

6. Had you heard of Descemetorhexis without endothelial keratoplasty (DWEK) as a surgical procedure, prior to this survey?

Options: Yes, No.

7. Have you attended a training course to learn Descemetorhexis without endothelial keratoplasty (DWEK)?

Options: Yes, I plan to attend. No, I would like to. No, I plan to self-teach this technique. Yes, I have attended. No, I do not plan to learn this technique. 

8. If you are already performing Descemetorhexis without endothelial keratoplasty (DWEK), please estimate what percentage of your corneal transplant cases for Fuchs endothelial corneal dystrophy (FECD) were DWEK. If you are not currently performing DWEK, please write 0.  

Short answer free text response.

9. What is your opinion on the role of Descemetorhexis without endothelial keratoplasty (DWEK) for the management of Fuchs endothelial corneal dystrophy, based on the current level of evidence?

Options: Promising technique with RHO kinase inhibitor, Promising technique without RHO kinase inhibitor, Not a practical solution, Other. 

10. Would further RCT evidence comparing Descemetorhexis without endothelial keratoplasty (DWEK) to DMEK and DSEK be of benefit?

Options: Very beneficial, Moderately beneficial, Somewhat beneficial, Not beneficial, Unnecessary. 

11. Would you or your trust be interested in participating in an RCT comparing Descemetorhexis without endothelial keratoplasty (DWEK) with DMEK? (If you would be interested in participating and are happy to be contacted, please also add your email to the 'other' box)

Options: Yes, No, Other. 

12. What is the likelihood that you would change your keratoplasty/without keratoplasty technique based on new RCT results?

Options: Very likely, Moderately likely, Somewhat likely, Not likely.

13. Would you consider Descemetorhexis without endothelial keratoplasty (DWEK) even if RHO kinase inhibitors are not accessible in your trust?

Options: Yes, No. 

14. Any additional comments.

Long answer free text response. 

Appendix 2: Google survey responses (as summarised in Table 1)

**Table 1:** Appendix 2-Google survey responses

Timestamp	1. Please select the region of your most recent practice (please select all that apply)	2. Which trust do you practice in NHS?	3. Please indicate your specialty job title	4. Please indicate how many years you have been practicing in cornea	5. What is your current practice for the management of Fuchs endothelial corneal dystrophy? (please select all that apply)	6. Had you heard of Descemetorhexis without endothelial keratoplasty (DWEK) as a surgical procedure, prior to this survey?	7. Have you attended a training course to learn Descemetorhexis without endothelial keratoplasty (DWEK)?	8. If you are already performing Descemetorhexis without endothelial keratoplasty (DWEK), please estimate what percentage of your corneal transplant cases for Fuchs endothelial corneal dystrophy (FECD) were DWEK. If you are not currently performing DWEK, please write 0.	9. What is your opinion on the role of Descemetorhexis without endothelial keratoplasty (DWEK) for the management of Fuchs endothelial corneal dystrophy, based on the current level of evidence?	10. Would further RCT evidence comparing Descemetorhexis without endothelial keratoplasty (DWEK) to DMEK and DSEK be of benefit?	11. Would you or your trust be interested in participating in an RCT comparing Descemetorhexis without endothelial keratoplasty (DWEK) with DMEK? (If you would be interested in participating and are happy to be contacted, please also add your email to the 'other' box)	12. What is the likelihood that you would change your keratoplasty/without keratoplasty technique based on new RCT results	13. Would you consider Descemetorhexis without endothelial keratoplasty (DWEK) even if RHO kinase inhibitors are not accessible in your trust?	14. Any additional comments
11/4/2023 12:00:35	Yorkshire and the Humber	Leeds Teaching Hospitals NHS Trust	Cornea consultant	14 years	Descemet membrane endothelial keratoplasty (DMEK), Descemet stripping (automated) endothelial keratoplasty (DSEK/DSAEK)	Yes	No, I plan to self-teach this technique	0	Promising technique with RHO kinase inhibitor	Very beneficial	Possibly	Somewhat likely	No	Need more evidence in this area
11/4/2023 12:00:49	South East	University Hospitals Sussex NHS Foundation Trust	Cornea consultant	18	Descemet membrane endothelial keratoplasty (DMEK), Descemet stripping (automated) endothelial keratoplasty (DSEK/DSAEK), penetrating keratoplasty	Yes	No, I plan to self-teach this technique	0	Promising technique with RHO kinase inhibitor	Very beneficial	Yes	Very likely	No	
11/4/2023 12:06:20	Yorkshire and the Humber, London	Calderdale and Huddersfield Royal Infirmary	Cornea consultant, Cornea fellow	4	Descemet membrane endothelial keratoplasty (DMEK), Descemet stripping (automated) endothelial keratoplasty (DSEK/DSAEK)	Yes	No, I plan to self-teach this technique	0	Promising technique with RHO kinase inhibitor, Not a practical solution	Very beneficial	Yes	Very likely	No	DWEK with Rhok inhibitors with significant results backed by RCT would be game changer in the field of cornea. Hundreds of thousands people would benefit, not only in the UK but all over the world.
11/4/2023 12:11:13	Yorkshire and the Humber	LTHT	Cornea consultant	30	I have retired from corneal surgery but would be practising DMEK if not	Yes	No, I do not plan to learn this technique	0	Promising technique with RHO kinase inhibitor	Very beneficial	Yes	Very likely	No	Patient selection seems very critical, needs a high quality specular microscope ?
11/4/2023 12:18:56	London	The Princess Alexandra	Cornea consultant	10	Descemet membrane endothelial keratoplasty (DMEK), Descemet stripping (automated) endothelial keratoplasty (DSEK/DSAEK)	Yes	Yes, I plan to attend	0	Not a practical solution	Very beneficial	Yes	Very likely	No	PRIVATE EMAIL
11/4/2023 12:20:36	South West	UHBW	Cornea consultant	6	Descemet membrane endothelial keratoplasty (DMEK)	Yes	Yes, I have attended	0	Promising technique with RHO kinase inhibitor	Moderately beneficial	Yes	Moderately likely	No	
11/4/2023 13:21:08	London	Barts Health NHS Trust	Cornea consultant	2	Descemet membrane endothelial keratoplasty (DMEK)	Yes	No, I plan to self-teach this technique	0	Promising technique with RHO kinase inhibitor	Moderately beneficial	Yes	Moderately likely	Yes	
11/4/2023 14:55:32	Yorkshire and the Humber	The Rotherham NHS Foundation Trust	Cornea consultant	4 years	Descemet membrane endothelial keratoplasty (DMEK), Descemet stripping (automated) endothelial keratoplasty (DSEK/DSAEK), penetrating keratoplasty	Yes	Yes, I plan to attend	0	Promising technique with RHO kinase inhibitor	Very beneficial	I am on career break	Moderately likely	No	
11/4/2023 14:58:09	London	Moorfields	Cornea consultant	6	Descemet membrane endothelial keratoplasty (DMEK)	Yes	No, I plan to self-teach this technique	0	Promising technique with RHO kinase inhibitor	Somewhat beneficial	No	Not likely	No	
11/4/2023 15:52:28	Yorkshire and the Humber	York	Cornea consultant	5 years	Descemet membrane endothelial keratoplasty (DMEK), Descemet stripping (automated) endothelial keratoplasty (DSEK/DSAEK)	Yes	Yes, I plan to attend	0	Promising technique with RHO kinase inhibitor	Very beneficial	Yes	Very likely	No	
11/5/2023 0:26:04	West Midlands	Sandwell and West Birmingham NHS Trust	Cornea consultant	5	Descemet membrane endothelial keratoplasty (DMEK), Descemet stripping (automated) endothelial keratoplasty (DSEK/DSAEK)	Yes	Yes, I plan to attend	0	A potential technique with RHO kinase inhibitor, but only suitable for selective cases with mild Fuchs endothelial dystrophy	Very beneficial	Yes	Very likely	No	
11/5/2023 8:41:02	Yorkshire and the Humber	Leeds Teaching Hospitals	Cornea consultant	6	Descemet membrane endothelial keratoplasty (DMEK)	Yes	No, I plan to self-teach this technique	0	Promising technique with RHO kinase inhibitor	Very beneficial	Yes	Very likely	No	NA
11/5/2023 9:44:23	Yorkshire and the Humber	Hull University teaching hospitals	Cornea consultant	3	Descemet membrane endothelial keratoplasty (DMEK), Descemet stripping (automated) endothelial keratoplasty (DSEK/DSAEK)	Yes	No, I plan to self-teach this technique	0	Promising technique with RHO kinase inhibitor	Very beneficial	Yes	Very likely	Yes	
11/6/2023 10:17:54	Yorkshire and the Humber	Leeds	Cornea consultant	5	Descemet membrane endothelial keratoplasty (DMEK)	Yes	No, I plan to self-teach this technique	0	Promising technique with RHO kinase inhibitor	Very beneficial	Yes	Very likely	No	
11/6/2023 14:35:27	East of England	University Hopsitals of Bristol and Weston	Cornea consultant	12	Descemet membrane endothelial keratoplasty (DMEK), Descemet stripping (automated) endothelial keratoplasty (DSEK/DSAEK), penetrating keratoplasty	Yes	Yes, I plan to attend	30	Promising technique with RHO kinase inhibitor	Very beneficial	I am not sure	Very likely	Yes	
11/7/2023 8:26:13	East of England	ESNEFT	Cornea fellow	1 year	Descemet membrane endothelial keratoplasty (DMEK)	Yes	Yes, I plan to attend	0	Promising technique with RHO kinase inhibitor	Very beneficial	Possibly	Moderately likely	No	
11/8/2023 6:31:34	North West	Northern Care Alliance NHS Foundation Trust	Cornea consultant	7	Descemet stripping (automated) endothelial keratoplasty (DSEK/DSAEK)	Yes	No, I plan to self-teach this technique	0	Not a practical solution	Very beneficial	Yes	Very likely	No	PRIVATE EMAIL
11/9/2023 8:54:58	London, South West	Private clinic	Cornea consultant	>5 years	Descemet membrane endothelial keratoplasty (DMEK)	Yes	No, I plan to self-teach this technique	0	Promising technique with RHO kinase inhibitor	Very beneficial	Yes	Very likely	Yes	
12/14/2023 18:06:43	East of England	ESNEFT	ST4	2 years	Descemet membrane endothelial keratoplasty (DMEK)	Yes	Yes, I plan to attend	0	Promising technique with RHO kinase inhibitor	Very beneficial	Yes	Somewhat likely	No	
12/21/2023 11:29:22	West Midlands	New Cross	Cornea consultant	20	Descemet membrane endothelial keratoplasty (DMEK), Descemet stripping (automated) endothelial keratoplasty (DSEK/DSAEK), penetrating keratoplasty	No	No, I plan to self-teach this technique	0	Not practicing	Very beneficial	Yes	Very likely	Yes	
1/3/2024 15:08:14	Yorkshire and the Humber	Sheffield	Cornea consultant	20	Descemet stripping (automated) endothelial keratoplasty (DSEK/DSAEK)	Yes	No, I plan to self-teach this technique	0	Promising technique with RHO kinase inhibitor	Moderately beneficial	No	Moderately likely	No	
1/8/2024 12:19:10	West Midlands	BMEC	Cornea fellow	2	Descemet membrane endothelial keratoplasty (DMEK), Descemet stripping (automated) endothelial keratoplasty (DSEK/DSAEK)	Yes	Yes, I plan to attend	0	Promising technique with RHO kinase inhibitor	Very beneficial	Needs discussion with the clinical lead	Moderately likely	Yes	
1/11/2024 21:39:01	Northern Ireland	NI	Cornea consultant	1	Descemet stripping (automated) endothelial keratoplasty (DSEK/DSAEK)	Yes	No, I plan to self-teach this technique	0	Promising technique with RHO kinase inhibitor	Very beneficial	No	Moderately likely	No	
1/19/2024 9:28:59	London	Kings College Hospital	Cornea consultant	20	Descemet membrane endothelial keratoplasty (DMEK), Descemet stripping (automated) endothelial keratoplasty (DSEK/DSAEK)	Yes	No, I plan to self-teach this technique	0	Promising technique with RHO kinase inhibitor	Moderately beneficial	No	Moderately likely	Yes	Only a very small % of patients would be suitable (i.e. those with reasonable endothelial cells in the periphery). Therefore it would be difficult to show it works in an RCT
3/28/2024 18:13:53	London	Not applicable TG	Cornea consultant	20+	Descemet membrane endothelial keratoplasty (DMEK)	Yes	No, I plan to self-teach this technique	Tried it once: not a success!	Promising technique with RHO kinase inhibitor	Very beneficial	No	Moderately likely	No	
3/29/2024 2:12:58	Oman	Army	Cornea consultant	25	Descemet membrane endothelial keratoplasty (DMEK), Descemet stripping (automated) endothelial keratoplasty (DSEK/DSAEK), Descemetorhexis without endothelial keratoplasty (DWEK), penetrating keratoplasty, RHO-associated kinase inhibitor eye drops, injection cell therapy, gene therapy	Yes	Yes, I have attended	40	Promising technique with RHO kinase inhibitor	Very beneficial	Yes	Very likely	Yes	
5/15/2024 12:58:07	East of England, West Midlands, London	NHS	Cornea consultant	2.5 years	Descemet membrane endothelial keratoplasty (DMEK), Descemet stripping (automated) endothelial keratoplasty (DSEK/DSAEK), penetrating keratoplasty, DALK, CAIRS, RHO-associated kinase inhibitor eye drops	Yes	Yes, I plan to attend	1	Not a practical solution	Somewhat beneficial	Yes, No	Moderately likely	No	more evidence is needed
5/15/2024 13:03:00	East of England	ESNEFT	Fellow	1	Descemet membrane endothelial keratoplasty (DMEK), Descemet stripping (automated) endothelial keratoplasty (DSEK/DSAEK)	Yes	No, I would like to	0	Promising technique with RHO kinase inhibitor	Very beneficial	Maybe	Moderately likely	Yes	
5/15/2024 18:14:04	East of England	ESNEFT	Fellow	One	Descemet membrane endothelial keratoplasty (DMEK), Descemet stripping (automated) endothelial keratoplasty (DSEK/DSAEK)	Yes	No, I would like to	0	Promising technique with RHO kinase inhibitor	Somewhat beneficial	Yes	Moderately likely	Yes	
5/20/2024 12:25:52	South East	Royal Berkshire	Trainee	2	Descemet membrane endothelial keratoplasty (DMEK), Descemet stripping (automated) endothelial keratoplasty (DSEK/DSAEK)	Yes	No, I plan to self-teach this technique	0	Not a practical solution, Not practical as challenging to get graft material if fails.	Somewhat beneficial	.	Somewhat likely	No	
5/20/2024 12:36:21	South East	Portsmouth	Cornea consultant	0.5	Descemet membrane endothelial keratoplasty (DMEK)	Yes	No, I would like to	0	Promising technique with RHO kinase inhibitor	Very beneficial	Yes	Somewhat likely	No	
5/20/2024 13:26:10	South East	Sussex Eye Hospital	Cornea consultant	1	Descemet membrane endothelial keratoplasty (DMEK), Descemet stripping (automated) endothelial keratoplasty (DSEK/DSAEK)	Yes	No, I would like to	NA	Promising technique with RHO kinase inhibitor	Moderately beneficial	Yes	Somewhat likely	No	
5/21/2024 10:12:17	Northern Ireland	Belfast HSC Trust	Cornea consultant	8	Descemet membrane endothelial keratoplasty (DMEK), Descemet stripping (automated) endothelial keratoplasty (DSEK/DSAEK), penetrating keratoplasty	Yes	No, I would like to	0	Promising technique with RHO kinase inhibitor	Very beneficial	Yes	Very likely	Yes	
5/21/2024 10:13:24	Northern Ireland	Belfast	Fellow	1	Descemet stripping (automated) endothelial keratoplasty (DSEK/DSAEK)	Yes	No, I would like to	0	Promising technique with RHO kinase inhibitor	Very beneficial	Yes, post fellowship	Moderately likely	No	
5/21/2024 10:16:02	Northern Ireland	Belfast trust	Cornea consultant	12	Descemet membrane endothelial keratoplasty (DMEK), Descemet stripping (automated) endothelial keratoplasty (DSEK/DSAEK)	Yes	No, I would like to	0	Promising technique with RHO kinase inhibitor	Very beneficial	Yes	Moderately likely	No	
5/22/2024 14:56:44	Mexico	Yes, public health	Cornea consultant	3 years	Penetrating keratoplasty	Yes	No, I would like to	0	Promising technique with RHO kinase inhibitor	Moderately beneficial	Yes	Moderately likely	Yes	
5/23/2024 14:28:03	Northern Ireland	Western trust	Cornea consultant	13	Descemet stripping (automated) endothelial keratoplasty (DSEK/DSAEK)	Yes	No, I would like to	0	Promising technique with RHO kinase inhibitor	Very beneficial	No	Moderately likely	No	
5/23/2024 14:28:55	Wales	Cardiff and Vale	Trainee	1	Descemet membrane endothelial keratoplasty (DMEK), Descemet stripping (automated) endothelial keratoplasty (DSEK/DSAEK), DWEK	Yes	No, I would like to	1	Promising technique without RHO kinase inhibitor	Moderately beneficial	Yes	Moderately likely	Yes	
